# Research on the Impact of the Emotional Expression of Kindergarten Teachers on Children: From the Perspective of the Class Micro-Power Relationship

**DOI:** 10.3389/fpsyg.2022.808847

**Published:** 2022-03-28

**Authors:** Min Liu, Qiong Wang

**Affiliations:** Graduate School of Education, Peking University, Beijing, China

**Keywords:** teacher emotions, children behavior, preschool education, micro-power theory, ethnography

## Abstract

During the preschool years, the socio-emotional responses children receive from interactions with teachers are incorporated into their own social behaviors. This is one of the key ways in which children acquire social and emotional skills. Based on field studies, it can be found that this learning process is not simple imitation of children, but of a more complex context of group interaction. To further clarify the impact of kindergarten teachers’ emotion on the sociometric status and behavior of 3–5 year-old children in their classes, the researchers chose a Montessori mixed-age kindergarten in Beijing as the field site and observed five classes within the kindergarten over a 2-month period in this ethnographic case study. The study found that the power gap between teacher and pupil spreads rapidly to all children in the classroom as a result of the teacher’s emotions, and even stimulates power stratification within the children. In addition, there are differences in the social behaviors between the children of different levels of power. As preschool children are in a critical developmental window when social knowledge is being accumulated and social skills are being acquired, using power relations within the kindergarten classroom as an entry point to analyze the impact of teachers’ emotions on children’s social behavior provides a new breakthrough for the professional development of early childhood education and the better achievement of educational goals.

## Introduction

Hochschild (1979) classified the types of labor as “physical labor,” “mental labor,” and “emotional labor.” Since then, more and more researchers have focused on the attributes of emotional labor in teachers’ work and its relationship to teachers’ professional development, particularly on the topics of teacher professionalism and burnout (Zhong, 2010; Liu et al., 2018). In addition to influences on their own professional lives, teachers’ emotions can have an important impact on the growth and development of their students, especially in the preschool years. In this phase, the social-emotional responses and related knowledge children receive from interactions with teachers can be incorporated into their own social behaviors that is one of the important ways in which children in early childhood learn social and emotional skills (Denham and Bassett, 2019).

Furthermore, with the introduction of the theory of Emotional Intelligence in the 1990s, the close relationship between emotion and cognition in terms of the mechanisms of production and action was further clarified (Salovey and Mayer, 1990). Based on this, researchers and practitioners advocated the promotion of Social and Emotional Learning (SEL) to promote the holistic, healthy, and sustained development of children and young people through the cultivation of social and emotional knowledge and skills (Zins and Elias, 2007). Since the beginning of the twenty-first century, the practice of SEL has spread globally and its educational value has now been widely recognized by researchers and practitioners both nationally and internationally (Zins et al., 2007; Durlak et al., 2011). With the accumulation of relevant research, it has been found that the preschool stage is an important window for children’s social and emotional development, a phase crucial for the development of children’s future learning qualities, interpersonal relationship building, and sound personality formation (Torres et al., 2015). “Emotional Socialization” is a key learning approach for children in this stage to acquire social emotion. The social-emotion responses and knowledge gained through interactions with parents, teachers, and other adults are incorporated into their own social behaviors (Denham, 1997; Denham and Bassett, 2019). Therefore, there is both theoretical and practical value in exploring how teachers’ emotions influence the development of children’s social behavior and related competencies from the meso perspective of the classroom community and the micro perspective of teacher–student interaction.

In this study, the major question was how the kindergarten teacher’s emotional expression affected the social behavior of children in class. This question is drawn not only from the existing studies mentioned above, but also from the researchers’ extensive field practice in kindergartens. During the field research in kindergarten, the researchers found that teachers used language as a medium to convey their emotions toward particular children; children were very sensitive to the teacher’s emotions, and thus imitating the teacher’s emotional expressions, and permeated them in the establishment of social relationships. For example, if children noticed that a teacher was constantly regulating a child with emotions, such as anger, they would often blame the child socially or be reluctant to establish a friendly social relationship with that child. The children who are often disciplined became the “social pits” of the group. This phenomenon indicated the power gap between children within a kindergarten class. This has inspired the researchers to understand this phenomenon through the lens of power relations, where the teacher’s emotions appear to accelerate the diffusion of power relations between teacher and pupil, and where other children are driven to emulate the behavior of the teacher, a person of higher power, which in turn affects the children’s own social behavior. To further clarify this research phenomenon, the study hopes to build on existing research and field observations, adopting classroom power relations, and the psychology of emotions as a theoretical basis to explore in depth the relationship between kindergarten teachers’ emotional expressions and children’s social behavior.

## Literature Review: Dynamic Power Relations in Kindergarten Classes

### Power Relations Within Children: Leadership

Parten (1933) alluded to a definition of child leadership as action-based, involving the act of directing others, and more specifically, leadership is referred as “when a child gives direction, command, order, request, or persuasion, etc., to other children over whom he has influence and from whom he gets cooperation and submission” (Fu, 1977, p. 13). Researchers assert that some children acquire the higher position of power and show the leadership in social activities. For example, the latest synthesis research by Chen (2021) reviewed the existing observation-method based empirical research on preschool children’s leadership at play, and found that the previous studies either dichotomized child leaders into “diplomat-bully,” “successful-unsuccessful leadership,” or labeling them as “dominator-director” according to their behaviors, and there are studies that categorized leadership styles specifically into dimensions and distinguished the types of children’s leaders according to the attribute values of children on each dimension. In addition, Chen’s reviewed the commonalities among preschool child leaders compared with their non-leader counterparts, including that child leaders were socially, linguistically, and cognitively more advanced, biologically older, more active in play participation, more likable by their peers, and socioeconomically more advantaged. Moreover, Chen suggested that child leadership may be attributable to the contextual influences, such as group dynamics and individual cultural experiences, which may be an important research direction but only a few studies have explored so far.

The power gap between preschool children observed by the researchers during fieldwork seems to show the presence of child leadership in the class: some children act as leaders, who occupy a commanding position in the class and direct their peers in the natural social activities. How dose these “little teacher” or “senior” appear? Based on field observations, the researchers found that their directing actions and higher ranking come out of imitation of their teachers, rather than fully influenced by factors, such as children’s developmental level, age, popularity, and socioeconomic status. It inspires us to ponder how come they have the opportunity to gain more power and are able to direct other children, and why they the imitate teachers’ behaviors. Distinct from the previous studies focusing on children’s characteristics, this research highlights the influence of teacher emotion as a contextual factor on children’s power ranking and corresponding social behaviors, which we think better reflects the dynamics of power relation in classes.

### Power Relations Under Negotiation Between Teachers and Children

The emergence of “little teachers” in the class means that teachers have transferred part of their power to children, actively or passively, allowing these children to acquire higher power status than the other peers. It reflects the transformation of power relations between teachers and children in the class. In a traditional class environment, the power relationship structure between teachers and students was top-down established, teacher-centered, and relatively stable. However, with the renewal of educational concepts and philosophy, child-centered pedagogy has been gradually prevailed, positioning children as active and competent agents in the present-day discourses of early childhood education (Wood, 2014; Nxumalo et al., 2018; Jobb, 2019). Under the child-centered pedagogy and educational philosophy, the power relations between teachers and children in the class shows a certain complexity and fluidity: Teachers and children dynamically negotiate and adjust their power relation, so that children have the opportunity to acquire part of the power that originally belongs to teachers in a traditional classroom.

The dynamic nature of power relations is one of the basic ideas of the micro-power theory of Michel Foucault (1926–1984). Foucault, as a leading contemporary philosopher, argued that wherever we are and whatever position we are in, we will be in a system of power constituted by the rules of reality. Foucault’s view of power emphasizes that modern power is diffused in every corner of society, every group, and that power is diffused in relations, networks, and fields. It is a non-subjective, non-centralized “disciplinary” mechanism, in which everyone in the network of power is being controlled while remaining to be the source of power. The classroom is a typical field of power determined by various types of power in which the teacher and the children are inevitably influenced. In traditional teacher-centered classroom, the external manifestation of this field of power is the teacher’s uninterrupted and continuous control over children’s physical activities through techniques, such as rigorous space allocation, time division, activity planning, and power programming, or what Foucault calls “the making of individual by discipline” (Foucault, 1979). Take traditional teacher-centered classroom as field of power, the legitimacy of power is displayed through the teacher’s authority, and the operation of power is mainly through discourse. This discursive power is formed by prescribing what each teacher and student should say, in what way, and how to say it. It can be argued that the operation of power in the classroom piggybacks on classroom discourse to control students, and that discourse is a way of releasing power and also a tool for its operation. While in modern child-centered classroom, teachers and students negotiate the power relationship through discourse. It is what Foucault calls “discourse as power” (Foucault, 1972).

According to the field observation in this research, due to the transformation of the current educational philosophy and the reconstruction of power relationship structure in the class, children as we called “little teacher” above have the opportunity to acquire additional power, resulting in the inequality of power status among children, and those in higher power status direct and instruct other children like what teachers do.

### Children’s Internal Pursuit of Power From the Perspective of Emotional Psychology

In addition to the external transformation of pedagogical philosophy and the changes of power relations, children’s internal pursuit of higher power ranking drives them to imitate teachers’ directing behavior and become leaders in the class. The emotional psychology from sociological perspective provides with the theory to explain how power relationship within a group influence the social behavior of its members. Theory of emotions on power-status, from Theodore Kemper, takes the changes in power status as the most common factor to trigger emotion. The members of different power status have different ways to express and experience emotion: Members in higher ranking are more likely to be angry and despise, while lower ranking are prone to be surprised and afraid (Kemper, 1990). In addition, the study from Lawler et al. (2000) shows that positive emotion ties people in the social relations. In other words, the urge and pursuits of each individual toward positive emotion naturally exists in social relations, which is an internal motivation to escalate power status.

Furthermore, given the principles of inter-group emotions and the inter-group assessment pathway in the formation mechanism, there may be a common consideration within the group based on shared interests rather than individual, resulting in a certain convergent emotion. Kenway and Fahy (2011) refer it as an “emotional resonance” (Emoscape). For instance, criticizing one child in a group activity may elicit anger from the whole class. This emotional communion in a particular spatial and temporal context may affect the social behavior and relationships of individuals in a particular group, e.g., blames or rejection toward the criticized child because of shared anger in a group. Therefore, there are often some followers around, who follow the child leaders to imitate teachers and direct other children.

Combined with the above literature review and our field work, this study makes a reasonable inference, that the kindergarten teachers’ emotional expression through discourse is a tool to release power relations in the class and a catalyst to enhance the speed and efficiency of resonant power relations. Brought out by teachers’ emotion embedded in discourse, children in the class quickly perceive the existence of power relations and adapt their social behaviors in response to their drive to pursue higher power status. Further, this research proposes the following questions: under the stimulative influence of teachers’ emotional expression behavior, what are the outcomes of power stratification for children in kindergarten classes? What are the typical social behaviors of children at different power status?

## Research Method

### Research Field and Participants

The research data come from a larger qualitative study in which the researchers conducted extensive participant observations from May 2019 to July 2021 in a Montessori kindergarten in Beijing, China. This larger study aimed to refine the instructional design principles of situated teaching for promoting children’s social and emotional knowledge and skills, and provide practical instructional guidance for the front-line teachers. An important work of the larger study is to observe and compare the children’s natural emotional socialization during the interactions with teachers, and the SEL process in the classroom intervention conditions.

Among data collected in the larger study, the observation data and teacher in-depth interviews used in this study were collected from December 2019 to January 2020. The researchers observed 5 classes in the kindergarten in a participant position. On an average, there were 15 children in each class, and the average age of the children in the five classes was 52.5 months (46.7% are girls; 53.3% are boys). Since the kindergarten is a for-profit private kindergarten, children who enter the field kindergarten generally come from a considerably higher socioeconomic background and their parents received good education (the yearly cost of the field kindergarten is about 140,000 RMB, while that in public kindergarten in Beijing is about 10,000 RMB per year).^1^ Each class has a foreign teacher, a Chinese AMS Montessori certified lead teacher (equivalent to the role of a homeroom teacher), an assistant teacher (responsible for assisting lead teacher, such as maintaining classroom orders and taking children to the toilet), and a life teacher (responsible for taking care of children’s daily life, such as preparing lunch and bedding), which is a relatively ideal teacher-to-student ratio for providing support to children. All teachers in the five classes were women, except for two foreign teachers who were men.

### Data Collection and Analysis

Specifically, the fieldwork process for this study included following steps. First, the researchers went to the kindergarten 1 day a week during the study period and entered the classroom to spend a day (from 8:00 to 15:00) in kindergarten with the children, such as breakfast in the morning when the children arrived, participation in Circle Time led by the foreign teacher, Montessori-style free work, as well as outdoor activities, lunch, naps, and afternoon teaching activities. Each day in the kindergarten classroom, the researchers videotaped the above activities with permission, and during observations, we recorded extensive fieldnotes by pen and paper. The researchers reviewed and collated the data collected on the same day and wrote a memo, thus ensuring that the field notes were abundant and authentic. In addition, the researcher interviewed the lead teacher of the class and observed to gain insight into the teachers’ understanding of the relationships within the class group and to exchange views on key events in the class. After 2 months of observation, the researchers obtained a total of more than 50,000 words of classroom observation and over 40,000 words of teacher interviews transcripts.

During and after the data collecting process, we used qualitative Software Program NVivo to encode and analyze the data to enter and participate in the lives of the research subjects and present a detailed, dynamic, and contextualized picture of people under study, to see things from the perspective of local and discover the “meaning” hidden behind the concrete situations (Chen, 2000; Fetterman, 2007). The main codes conceptualized during the data analysis stage included emotional expression, power stratification, little teacher, supervision and discipline, power regulation, power ebb, social avoidance, neutral, etc.

### Trustworthiness

Qualitative researchers should avoid the dangers to trustworthiness from multiple aspects, and the most helpful strategies include the following, that honestly introduce the research and build-up mutual trust with the participants, anonymize the field site and participants appearing in the to-be published research papers, provide reciprocal help to the participants as rewards, conduct field work rigorously in data collection and analysis, and etc. (Fetterman, 2007).

As mentioned above, the observation and interview data used in this research come from a larger qualitative study, and thus the researchers have already familiarized with the teachers and children in the field site and developed trust with them before entering the five classes. For example, the teachers are quite willing to consult us about problems they encounter in the teaching practice. As a result, the teachers agreed without hesitation for us to enter their classes and conduct observations after we introduced our research plan. Besides, as the kindergarten allows parents to attend classes and set up special observation seats in the classroom, we find no clear evidence that the children in the five classes were excessively affected by our presence. At the end of the 2-month observation, we shared our research findings and offered practical teaching suggestions for the teachers, with all private information were anonymized.

The data used in this research are collected from various sources, such as observation of the researchers, video recordings, and teachers’ interpretations revealed in the interviews. Data from the above sources valid each other triangularly, which ensures the quality of both the data and research.

## Findings of Study: Power Stratification and Social Performance of Children

In the chosen class, the teacher has the highest position of power within the class due to greater knowledge; so the disciplinary words or even reprimand from teacher can convey the disparity in power status between teacher and student. Moreover, the power relationship between the teacher and the reprimanded child is often accelerated by the expression of emotions, such as anger, prestige, and helplessness, which are transmitted to all children in the class. Children in the class are sensitive to the power relationships reinforced by negative emotions. Some children, driven by an inherent instinct for positive emotions, imitate the teacher’s behavior and take over some of the teacher’s power, acting like a “little teacher” in the class to supervise other children. They become the higher power within the group of children in the class. Most children are influenced by the emotional resonance and perceive the negative emotions conveyed by the teacher when disciplining other children, so they are more likely to agree with or ignore the “little teacher” when they discipline their peers. Children who are frequently named and criticized by the teacher are at the bottom of the hierarchy of power within the kindergarten classroom, and in addition to receive criticism from the teacher, they are also monitored and disciplined by the “little teacher” or even the “little teacher’s” supporters, i.e., children in the middle of the power hierarchy. When it comes to socializing with these children of lower hierarchy, other children may act the part or lack of interest to do so; thus over time, the lower-ranking children in the power relationship may become “socially deprived.”

### Higher Ranking Children: “Little Teacher” or “Senior”

In the kindergarten classroom, teachers actively or passively delegate some of their power to children. Active delegation includes teachers calling on children to monitor each other’s behavior, and teachers explicitly asking children in the class to help monitor the behavior of particular children. The latter being less common in kindergartens nowadays because it hinders an egalitarian and friendly atmosphere in the classroom. Specifically, the teacher establishes a certain amount of peer supervision within the class through discursive behavior, for example, by first introducing the social rule of taking turns in using teaching materials within the class and informing children that if a peer breaks this rule and there is an argument, the child can correct the other child’s behavior or raise hand to tell teacher. After the verbal introduction of the rule, the teacher reinforces the rule mainly through more physical behavior, for example, when a child acts out of line, the teacher immediately stops the child, and the emotions expressed by the teacher when stopping the child, such as anger, disappointment, and helplessness, magnify the power relationship between the teacher and the pupil, thus stimulating some children to imitate the social behavior of the teacher, a higher power, in pursuit of a positive emotional experience. As a result, the peer monitoring mechanism within the kindergarten classroom gives rise to children in the positions of power, such as the “little teacher” or “senior.”

According to teacher interview, “Children know how to treat others accordingly. For example, the child in our class called King Kong sometimes thinks outside the box and cannot express himself clearly. He is used to imitating the behavior from cartoon or tale characters. When doing so, he cannot control himself properly, and others may get hurt. Teachers sometimes are relatively strict with him. For a while I would get angry and irritable and would stop King Kong’s behavior very sternly. Soon, Tang Xin would often say, “King Kong, put this there” or “King Kong, you can’t do that” and so on. This may be our understanding as adults, imposing our own ideas on Tang Xin; but what we see is that Tang Xin will only “instruct” a few children. This is also normal in Montessori kindergarten classes, where the child will imitate adult and can tell who the teacher likes and dislikes, and she will then condescend to instruct the children in the class who often don’t follow the rules.”

Another example is Hao Hao: “Hao Hao in my class is capable of imitation. Besides, he is the oldest in class, who often considers himself as a little teacher. I would often ask students, “Why are you doing that again?” “Didn’t I tell you not to do that?” “I’ve called you several times, did you hear me?” Then, I found that Hao Hao repeating all these words, and even more fiercely. He would say to other children, “I’ve warned you, can’t you hear me?” “Can’t you see I’m here?” His tone is full of loathe like an adult. I found him a shadow of me: Every time I was impatient and irritated, he would be the same, quite explicitly. And Hao Hao doesn’t say this to all the children. For example, there is a child in the class called Fan Shu, and when he is with other children, he will show kindness by jumping on someone’s shoulder or patting them on the head, but other children may interpret this as Fan Shu hitting me. When there are more arguments like this, we sometimes feel helpless and say, “Why do you have to use such a move when you can behave?” Hao Hao would often criticize Fan Shu or come to me and say things like “Fan Shu is hitting someone again.”

During the peer socialization process, Tang Xin and King Kong, Hao Hao and Fan Shu do not interact equally, as Tang Xin and Hao Hao often take on the role of “senior” or “little teacher,” as the teachers say, to “instruct” or even “discipline” King Kong and Fan Shu. From the perspective of child developmental psychology, this social behavior of Tang Xin and Hao Hao is a social reference and imitation of the teacher’s behavior; from the theories of classroom power systems and emotional psychology, a quest for a higher position of power. In the kindergarten classroom, there are often children who actively share some of power from teacher, such as Tang Xin, who uses teaching and instructive power of teacher’s. This additional power gives children like “Tang Xin and Hao Hao” a higher and closer position of authority to the teacher than other children. In fulfilling the role of “little teacher” or “senior,” these children often experience positive social experiences when exercising the power of their roles, as evidenced by the expressions of dislike and anger toward the inferior, and often display emotions of pride, smugness, satisfaction, and happiness when they “report” the inferior’s rule-breaking behavior to the teacher.

It is important to note that within the class, there are children, such as Tang Xin and Hao Hao who actively share the teacher’s authority, and teachers may also tacitly or actively relinquish some of their authority to these students. For example, in the story of Hao Hao and Fan Shu, the teacher initially behaves in a way that tacitly allows Hao Hao to be the “little teacher,” but later, the teacher notices that Hao Hao is “abusing” the power, over-supervising and directing Fan Shu, and even involving attack-ish behavior in social performance, then teacher limits the power of Hao Hao.

As the headteacher said, “Four teachers in our class all disciplined Fan Shu. Hao Hao came to me every day to tell Fan Shu off, saying that Fan Shu hit him on the head again on the school bus and so on. I thought Hao Hao was a bit crossing the line, so I told him that he is already five years old and has to tell apart accidental touching and hit on purpose. I told him to tell the difference for several times, but you would find him smug every time he tells Fan Shu off.”

In the kindergarten classroom, it can be inferred that whether the “little teacher” who is in a higher power ranking becomes a role model or a “prickly king” among the children, depends on the teacher’s power regulation strategies. For example, if the homeroom teacher of Fan Shu and Hao Hao has acquiesced in the transfer of power to Hao Hao, Hao Hao may monitor within the children, contributing positively to the emergence of Hao Hao’s sense of self and the effectiveness of classroom management. However, when power is abused, such healthy supervisory behavior has turned into social aggression, teachers need to adopt strategies to limit the power of the “little teacher” and rebalance power relations within the classroom.

### Children in Lower-Ranking: “Self-Adjuster at the Ebb”

In the kindergarten classroom, the “little teachers” and their supporters who share some of the teacher’s power may either monitor and discipline children, such as King Kong and Fan Shu, who are often criticized by the teacher, as Tang Xin and Hao Hao does, or they may avoid children, such as Yi Yi and Ruyi, described below, and “socially isolate” these children. In the power field of the kindergarten classroom, the “little teacher” and their advocates can discipline or isolate children, such as King Kong, Yi Yi, and Ru yi, while the latter are passive recipients despite their willingness. In this research, the latter are referred to as the low-power rankers within the kindergarten classroom, or “power pit.” In the context of power relations, the low-power rankers are under pressure on social performance, as other children may be less inclined to socialize with the criticized child, reinforced by group emotions. In the long run, the lower-power rankers are likely to become the “social pits” of the kindergarten class. During the fieldwork, fortunately, the researchers found that even in power and social pits, kindergarten children have already developed certain social behavioral strategies to regulate the negative social experiences of being in a low power position.

During participating observation in the kindergarten class, the researchers found that the 4-year-old boy, Yi Yi, had adopted the social strategy of interacting more closely with the teacher, facing the pressure of his low power ranking and social puddle. According to in-depth interviews with teacher, Yi Yi’s interests were very clear. He had a strong interest in books and words, and if the classroom activity was not in his interest, “Yi Yi would not sit down at all.” For example, he always fails to put the finished picture books back on the shelf. To help him establish a behavioral code, the teacher will say to him, “If you don’t put the books back on the shelf, you can’t read them today” and sometimes if he keeps disobeying or refuses to communicate with the teacher, the teacher will scold him with anger. As the teacher said, “sometimes my facial expression has changed, but I won’t do anything more than keep urging the child to do what he should do.”

Ru Yi, a boy in another class, faces similar situation. Ru Yi is often behind schedule. His procrastination usually “ignites” teacher’s negative emotion. Teacher needs to accomplish daily class activity as kindergarten requires, and takes care of every child in class, so it is hard for teacher to fully engage in helping these slow starters. Teacher may feel “anxious and a sense of inability” with Ru Yi. Other kids in class will not supervise and regulate Yi Yi and Ru Yi as “little teachers” do, but they show little willingness in interaction, “kind of avoiding him,” as the teacher comments. From the perspective of the teacher, social avoidance is impairing to the development of children. “With the group avoidance, you do not develop awareness; without awareness, you will not be socializing; without socializing, nothing goes further.” During class observation, the researchers found that these kids in social and power pits will adopt adjusting strategy to make up for the vulnerability.

As recorded during classroom observation, “After 9 a.m. Circle time, Yi Yi walked toward plants dial. The teaching material was occupied, so Yi Yi took out two planet balls. Instead of inviting Yi Yi together, the boy who was using that plants dial took Yi Yi’s planet balls. As a result, Yi Yi tried to seek help. He turned to homeroom teacher first without saying anything but pointed at that dial. Teacher did not respond at once, so Yi Yi went back to the dial. There were two more boys by the dial that time and told Yi Yi to go away. Yi Yi had to go to the foreign teacher, but he did not get any assistance either. Yi Yi went on third strike. The homeroom teacher required Yi Yi to express his needs verbally, then took Yi Yi to join others at the dial and learnt about planets.” …… “At 9:13 a.m., Yi Yi would like to attend another table for plasticine activity. However, his participation was interrupted. Assistant tutor told him to put planet dial away before attending. Yi Yi returned to the dial, while new participant was playing the dial and prevented Yi Yi from organizing. Yi Yi turned to teacher for help by nuzzling up against the teacher without a word. Teacher then led children including Yi Yi to do the plasticine activity. After the activity, Yi Yi held teacher’s hand and played the planet dial with teacher. With the assistant teacher gone, Yi Yi followed homeroom teacher to check out what the teacher was doing; then he checked on the foreign teacher. He was merely observing without participation.”

During observation, other children in class do not interact with Yi Yi closely. On the contrary, Yi Yi is refused and rejected by fellow students. Yi Yi does not engage actively. This is an indirect evidence of his awareness toward his position in this power field—the power pit. Being in the power and social pit, Yi Yi and Ru Yi display similar social behavioral strategy: they take teacher as major social objects. To Yi Yi and Ru Yi, rather than being disciplined or isolated by peers and acknowledge their inferior position, it is a better choice to interact with the teacher. Teacher is the authoritative high ranker, which is more easily to accept than “little teacher.” Teacher offers less negative emotion experiences. In addition, teachers in kindergarten often pay special attention to kids with problem behaving. They even sit with those kids for better regulation. In a word, the reason that these kids prefer interaction with teacher is both internally and externally driven. Some kindergarten teachers think that lower rankers are not definitely inferior to high rankers in cognitive and socialization development. For example, Yi Yi and Ru Yi get more direct instruction when interacting with teachers. The intention of this study is not to encourage negative emotional expression from kindergarten teacher. In other words, students have the ability to understand teacher’s negative emotion within a reasonable range. The negative emotional expression may not interfere with setting up quality interaction between teacher and student and the opportunity for students to acquire positive development.

### Children in the Middle Power Ranking: “Supporter of Little Teacher,” “Striker,” and “Neutral”

During field trip in the kindergarten, the researchers found that attention was easily attracted to certain kids while failed to notice some other kids even for a whole day. In other words, the sense of “being” varies from kid to kid. “Little teacher,” the higher power ranker, or lower ranker were more eye-catching to the observer. On the other hand, some kids participated all the class activities with mediocre performance, being hard to show their feature clearly. The researchers went over notes on class observation and teacher interview repeatedly but failed to find much material related to these kids with mediocre performance, both on their power ranking and social behavior. The field trip is not enough for the researchers to establish clear vision toward these kids. To enrich the resource, the researchers went back to the kindergarten. Combined with the existing class videos, researchers were able to take a closer look at the social behavior of these kids. The finding of this research is that these kids can be divided into three types: “supporter of little teacher,” “striker,” and “neutral.”

First, with the impact of emotional resonance, the negative emotion given out by teacher to designated children would diffuse to the whole class. Every child within the class would sense it and thus forming the similar negative emotion like the teacher. As stated above, part of the class would sharply identify the power difference between teacher and students, which is signified by negative emotion. To pursue more positive emotional experience in the group, they would play a role of higher power ranking as “little teacher” and “senior.” They are not only “little teacher” but individuals of more influence in the class. The rest of class react differently under the influence of “little teacher,” and they will generate various psychological experience and social behavior, which are related to the characteristic of “little teacher.” If a popular kid acts as the “little teacher,” the others would tend to be attracted and impelled to agree with “little teacher”; otherwise, they would possibly ignore or even strike their position in a higher power ranking.

Besides, there is a group of “neutrals” exist in the middle ranking. For example, Meng Meng and Tian Tian are criticized frequently, because they almost always fail to concentrate in class and disturb the class discipline. Most kids are able to sense teacher’s negative feeling toward Meng Meng and Tian Tian, and they choose to ignore them and avoid social interaction.

As recorded during classroom observation, “when the teacher was giving a lecture on Minor Cold, Meng Meng crawled under the desk, passing Xiao Jiu, Xiao Yi, and Kai Rui. The three of them all felt the disturbance, but they only took a glance and did nothing to stop him. Nor did they express specific feelings. Meanwhile, Tian Tian swiped his saliva onto the desk and said that his saliva smells as fragrant as his foot. Assistant teacher tried to stop him several times, however, Tian Tian stayed the same. Xiao Yi looked at Tian Tian without uttering a word.”

In a kindergarten class, a large group of children are “neutral,” such as Xiao Jiu and Xiao Yi. Although they feel the power relation under signified negative emotion, they are not voluntarily sharing teacher’s power as Tang Xin and Hao Hao do—being the “little teacher” and higher power ranker. They face no social pressure, such as Fan Shu and Yi Yi in the power pit, either. These kids selectively ignore and avoid power stratification. They seldom socialize with “little teacher” and lower rankers. With most of teacher’s attention going to those two roles, they have more chances to play with other similar “neutrals.” They are in the middle of power relationship, and more stable in expression emotion.

## Conclusion and Discussion

This study collected and analyzed rich ethnographical data to explore the influence of teachers’ emotion on the preschool children’s social behaviors. First, the research reviewed the literature about children leadership and power relations in class, to explain both the external and internal mechanism of how teachers’ emotion influences children’s power relation. Moreover, grounded by classroom observations and field work, we further clarify and elaborate the power stratification of children in kindergarten classes and the typical social behaviors of children in different power rankings.

Specifically, under the stimulation of teachers’ emotional expression, three power levels, high, medium, and low, emerged within the children group. Among them, some children incline to imitate teachers’ directing actions and become “little teacher” of a higher power position in the class, driven by their internal pursuit of better emotional experience and empowered in modern child-centered classrooms. Those children who are in a higher power ranking can either become a role model and leader with a sense of autonomy, or an annoying “prickly king,” depending partly on the teachers’ power regulation strategies. The children of higher ranking and their advocates tend to discipline children who are often criticized by the teacher, while the latter are passive recipients despite their willingness and they may even suffer from social avoidance under the effect of intragroup emotional resonance. But fortunately, the children of lower ranking show certain initiative to compensate for their scarce social interactions with peers, such as seeking help and interacting with teachers more actively and frequently. Between children of higher and lower ranking, there are also “supporter of little teacher,” “striker,” and “neutral” children in the middle. Most of them frequently socialize with the similar middle, and their emotional experiences and expressions seem to be more stable.

## Implications for Pedagogical Practice

The finding of this study would enlighten teachers to transfer part of their power to student appropriately, to regulate power relationships in class strategically. It is beneficial to activate the children’s autonomy and enhance ability to run the class by themselves. In conventional education concept, teacher holds almost all the power to teaching while students can only cooperate passively. But now this power field form has already changed. In modern educational context, with the popularity of ideological trend of humanism, “student-centered” had become the consensus of educational researchers and practitioners. In this study, even kindergarten children acquire willingness and ability to share the power, which relatively means that class manipulated by the teacher has transformed into semi-autonomous. The development of all students is led by themselves, while teachers are there for necessary guidance and assistance. Teachers need to be aware of how their emotion influence students in organizing the class. In class activities, they need to cultivate students’ autonomy, interfere timely when the power stratification occurring within students as a result of power transfer. Teachers will need to adopt certain strategy to limit the power abuse of high ranker, help low ranker to adjust social relationship, and create loving, united atmosphere in class.

To further elaborate, although teacher’s emotion affects the power relationship and social behavior of students, it is not necessary to require teachers to perfectly disguise their emotion, especially kindergarten teachers. According to previous research, depressing or disguising emotion are detrimental to teacher, which includes even more fierce emotional expression and lower satisfaction on interpersonal relationship (Rivers et al., 2007). The downward working satisfaction and happiness are particularly eye-catching, causing worse occupational burnout (Chang, 2009; Lechuga, 2012).

What we lose in hake we shall have in herring. In addition, the study finds that students in lower power rank develop their social regulatory strategy, such as enhancing interaction with teacher, which is also helpful for the development of children social ability. Additionally, moderate emotional expression from teacher works for establishing positive teacher-student relation and the cognitive development of students (Oplatka, 2011). In conclusion, teacher is encouraged to break the dilemma of “being the true self” and “faking themselves” (Li, 2018) from a perspective of effective teaching. The negative emotional expression may reinforce power relationship in class, and stimulate the power stratification within students. However, the cutting out natural and reasonable emotional expression of teacher is not a necessity. Offering scientific instruction on how to regulate power relationship within class and targeted intervention on children in different power rankings will be a more humanistic and effective way.

A class is the basic teaching unit in school, the daily context where teacher teaches and students study. Class could be regarded as a special social group, and the specialty lies in power classification and the transfer of its members will influence the realization of educational purpose in class activities. Children are in the critical development phase to accumulate social knowledge and master social skills, especially in preschool phase. The study takes internal power relationship in kindergarten class as a point to analyze how teachers’ emotion affect children’s social behavior. This study develops an in-depth understanding on the need of a class, contributes to the new break point in development of preschool profession, and better realization of educational aims.

## Implications for Research Directions

The research finding inspires future research to highlight the influence of contextual factors when exploring the forming mechanisms of children’s leadership and power relations in classes.

As Chen (2021) mentioned in the synthesis research, 3–5 year-old children’s leadership are considered to be partly attributable to the contextual factors, such as group dynamics. Inspired by existing literature and field observation, this study clarifies how teachers’ emotional expressions and teacher-children power relations influence children’s directing actions and social behaviors. To further expand the understanding of children’s leadership, the influence of more micro and contextual factors, especially children’s gender, and their functional mechanism should be explored in the future. In addition, both emotional expressions and power stratification in classes affected by social and cultural background to a certain extent (e.g., Wong, 2016; Ip et al., 2021), so it remains to be answered whether the research findings are still tenable after leaving the China’s educational and cultural soil. It is worthy for researchers to conduct further research, and this may become a breakthrough for understanding specific educational phenomena from a cross-cultural perspective.

Teacher-student relationship and their interactions are dominant topics in education field, which is regarded as an important entry point to study children’s cognitive, social, and emotional development (Meškauskienë, 2017; Wang, 2020; etc.). This study emphasizes the influence of teachers’ emotional expressions on preschool children’s social behaviors, and inspires future studies to focus on more profound and distal outcomes. As mentioned in by a teacher interviewed in this study, for example, the children of lower ranking can take the initiative to interact with teachers to compensate for their scarce interactions with peers, and as a result they get more targeted guidance from the teacher. Besides, based on the teacher’s observation, these children are likely to have a more positive cognitive development than other children. Surely, more subsequent empirical studies are needed to verify the hypothesized relation between children’s power ranking and their cognitive development.

## Data Availability Statement

The original contributions presented in the study are included in the article/supplementary material, further inquiries can be directed to the corresponding author/s.

## Ethics Statement

The studies involving human participants were reviewed and approved by the Peking University. Written informed consent to participate in this study was provided by the participants’ legal guardian/next of kin.

## Author Contributions

ML conducted the participant observation and teacher interviews in the field Kindergarten, and through the field work, put forward the major idea of this study. QW directed ML in the entire conceptual framing and writing logic, and contributed to writing and editing this manuscript. Both authors contributed to the article and approved the submitted version.

## Conflict of Interest

The authors declare that the research was conducted in the absence of any commercial or financial relationships that could be construed as a potential conflict of interest.

## Publisher’s Note

All claims expressed in this article are solely those of the authors and do not necessarily represent those of their affiliated organizations, or those of the publisher, the editors and the reviewers. Any product that may be evaluated in this article, or claim that may be made by its manufacturer, is not guaranteed or endorsed by the publisher.

## Footnotes

1 Data retrieved from: http://www.moe.gov.cn/jyb_xwfb/xw_zt/moe_357/jyzt_2019n/2019_zt4/bjx/mtjj/201905/t20190507_380923.html

## References

[B1] Chang M. L. (2009). An appraisal perspective of teacher burnout: examining the emotional work of teachers. *Educ. Psychol. Rev.* 21 193–218. 10.1007/s10648-009-9106-y

[B2] Chen J. J. (2021). Leadership at play in preschool children: a systematic synthesis of nearly nine decades of research. *Early Educ. Dev.* 1–26. 10.1080/10409289.2021.198803635082478

[B3] Chen X. M. (2000). Qualitative research methods and social science research. *Beijing: Edu. Sci. Publ. House. Page* 25:391.

[B4] Denham S. A. (1997). “When I have a bad dream, mommy holds me”: preschoolers’ conceptions of emotions, parental socialisation, and emotional competence. *Intern. J. Behav. Dev.* 20 301–319. 10.1080/016502597385351

[B5] Denham S. A. Bassett H. H. (2019). Early childhood teachers’ socialization of children’s emotional competence. *J. Res. Innov. Teach. Learn.* 12 133–150. 10.1108/JRIT-01-2019-0007

[B6] Durlak J. A. Weissberg R. P. Dymnicki A. B. Taylor R. D. Schellinger K. B. (2011). The impact of enhancing students’. *Child Dev.* 82 405–432. 21291449 10.1111/j.1467-8624.2010.01564.x

[B7] Fetterman D. M. (2007). *Ethnography: Step By Step.* Chongqing: Chongqing University Press, 145. trans G Jianhua.

[B8] Foucault M. (1972). *The Archaeology of Knowledge and the Discourse on Language (1st American ed.).* New York, NY: Pantheon Books.

[B9] Foucault M. (1979). *Discipline and Punish: The Birth of the Prison.* New York, NY: Vintage Books, 193. trans Y Yuanying and L Beicheng.

[B10] Fu V. R. (1977). A Nursery School Leadership Observation Schedule and a Nursery School Leadership Scale. ERIC Document Reproduction Service, No. ED 134601. https://files.eric.ed.gov/fulltext/ED134601.pdf (accessed February 2, 2022).

[B11] Hochschild A. R. (1979). Emotion work, feeling rules, and social structure. *Am. J. Sociol.* 85 551–575.

[B12] Ip K. I. Miller A. L. Karasawa M. Hirabayashi H. Kazama M. Wang L. (2021). Emotion expression and regulation in three cultures: Chinese, Japanese, and American preschoolers’ reactions to disappointment. *J. Exp. Child Psychol.* 201 104972–104972. 10.1016/j.jecp.2020.104972 32919326 PMC7583664

[B13] Jobb C. (2019). Power, space, and place in early childhood education. *Can. J. Sociol.* 44 211–232. 10.29173/cjs29596

[B14] Kemper T. D. (1990). “Social relations and emotions: a structural approach,” in *Research Agendas in the Sociology of Emotions*, ed. Kemper T. D. (Albany: SUNY Press), 335.

[B15] Kenway J. Fahy J. (2011). Public pedagogies and global emoscapes. *Pedagog.: Int. J.* 6 167–179.

[B16] Lawler E. J. Thye S. R. Yoon U. (2000). Emotion and group cohesion in productive exchange. *Am. J. Sociol.* 106 616–657. 10.1353/cpr.2018.0021 29755049 PMC6280975

[B17] Lechuga V. M. (2012). Emotional management and motivation: a case study of underrepresented faculty. *New Dir. Inst. Res.* 155 85–98.

[B18] Li H. Y. (2018). Real or fake expressing emotions? ———psychological process analysis of teachers’. *Emot. Work Teach. Educ. Res.* 30 103–109.

[B19] Liu D. Jiao R. K. Wang H. L. (2018). The relationship between preschool teachers’ emotional labor strategies and burnout: an application of latent profile analysis. *Psychol. Dev. Educ*. 34 104–111.

[B20] Meškauskienë A. (2017). The influence of educational teacher- learner interaction on adolescent self-esteem. *Pedagogika* 126 115–129. 10.15823/p.2017.23

[B21] Nxumalo F. Vintimilla C. D. Nelson N. (2018). Pedagogical gatherings in early childhood education: mapping interferences in emer- gent curriculum. *Curric. Inquiry* 48 433–453.

[B22] Oplatka I. (2011). “Emotion management and display in teaching: Some ethical and moral considerations in the era of marketization and commercialization,” in *Advances in Teacher Emotion Research*, eds Schutz P. A. Zembylas M. (New York, NY: Springer), 55–72.

[B23] Parten M. B. (1933). Leadership among preschool children. *J. Abnorm. Soc. Psychol.* 27 430–440. 10.1037/h0073032

[B24] Rivers S. E. Brackett M. A. Katulak N. A. Salovey P. (2007). Regulating anger and sadness: an exploration of discrete emotions in emotion regulation. *J. Happiness Stud.* 8 393–427. 10.1080/02699931.2019.1687426 31690211 PMC7198354

[B25] Salovey P. Mayer J. D. (1990). Emotional intelligence. *Imagin. Cogn. Pers*. 9 185–211.

[B26] Torres M. M. Domitrovich C. E. Bierman K. L. (2015). Preschool interpersonal relationships predict kindergarten achievement: mediated by gains in emotion knowledge. *J.Appl. Dev. Psychol.* 39 44–52. 10.1016/j.appdev.2015.04.008 27630379 PMC5019579

[B27] Wang A. J. (2020). Review and reflection on student-teacher relationship researches in China. *Soc Sci. Front.* 230–239.

[B28] Wong M. (2016). Teacher-student power relations as a reflection of multileveled intertwined interactions. *Br. J. Sociol. Educ.* 37 248–267. 10.1080/01425692.2014.916600

[B29] Wood E. A. (2014). Free choice and free play in early childhood educa- tion: Troubling the discourse. *Int. J. Early Years Educ.* 22 4–18.

[B30] Zhong C. Y. (2010). Design and implementation of emotional labor literacy training for teachers in pre-service stage. *Educ. Res. Exp*. 62–66.

[B31] Zins J. E. Bloodworth M. R. Weissberg R. P. Walberg H. J. (2007). The scientific base linking social and emotional learning to school success. *J. Educ. Psychol. Consult*. 17 191–210. 10.3389/fpsyg.2019.01885 31496971 PMC6712083

[B32] Zins J. E. Elias M. J. (2007). Social and emotional learning: Promoting the development of all students. *J. Educ. Psychol. Consult.* 17 233–255.

